# Factors of Agitation in Older Chinese With Cognitive Impairment in Long-Term Care Facilities: A Multilevel Analysis

**DOI:** 10.1093/geroni/igaf122.694

**Published:** 2025-12-31

**Authors:** Kaipeng Wang, Fei Sun, Xiang Gao

**Affiliations:** University of Denver, Denver, Colorado, United States; Michigan State University, East Lansing, Michigan, United States; Huazhong University of Science and Technology, Wuhan, Hubei, China (People’s Republic)

## Abstract

Agitation among older residents with cognitive impairment is a common challenge in long-term care facilities. However, systematic investigations into contributing factors of agitation of older Chinese residents with cognitive impairment in long-term care facilities remain limited. This study examines individual, family, staff, and facility-level factors associated with agitation in older Chinese with cognitive impairment in long-term care facilities. We collected individual and family data from 266 older adults with cognitive impairment (Mini-Mental State Examination (MMSE) score < 24) and facility staff members across 19 long-term care facilities. Data on staff and facility characteristics were ascertained from direct care workers (DCWs) and facility administrators. Results of linear mixed-effects models show that higher education levels and poorer psychological adjustment to the facility were associated with a higher level of agitation. In contrast, residents with better cognitive functioning and those who called family members at least once a week exhibited lower aggression. At the facility level, a higher mean of DCWs’ perceived organizational support and the presence of at least one DCW on a permanent contract were associated with lower agitation. These results underscore the complex influence of individual, family, staff, and facility factors on agitation among older Chinese residents. Interventions aimed at strengthening older resident family connections and enhancing DCW support systems may be critical in mitigating agitation and improving the well-being of this population.

